# Texture-Induced Strain in a WS_2_ Single Layer to Monitor Spin–Valley Polarization

**DOI:** 10.3390/nano14171437

**Published:** 2024-09-03

**Authors:** George Kourmoulakis, Antonios Michail, Dimitris Anestopoulos, Joseph A. Christodoulides, Manoj Tripathi, Alan Β. Dalton, John Parthenios, Konstantinos Papagelis, Emmanuel Stratakis, George Kioseoglou

**Affiliations:** 1 Institute of Electronic Structure and Laser, Foundation for Research and Technology—Hellas, 71110 Heraklion, Greece; geokourm@iesl.forth.gr (G.K.); stratak@iesl.forth.gr (E.S.); 2 Department of Materials Science and Engineering, University of Crete, 70013 Heraklion, Greece; 3 Department of Physics, University of Patras, 26504 Patras, Greece; antmichail@upatras.gr; 4 Institute of Chemical Engineering Sciences, Foundation for Research and Technology-Hellas, Stadiou Str. Platani, 26504 Patras, Greece; anestopd@gmail.com; 5 Naval Research Laboratory, 4555 Overlook Ave. SW, Washington, DC 20375-5320, USA; joseph.christodoulides@nrl.navy.mil; 6 Department of Physics and Astronomy, University of Sussex, Brighton BN1 9RH, UK; m.tripathi@sussex.ac.uk (M.T.); a.b.dalton@sussex.ac.uk (A.B.D.); 7 School of Physics, Department of Solid-State Physics, Aristotle University of Thessaloniki, 54124 Thessaloniki, Greece; 8 Department of Physics, University of Crete, 70013 Heraklion, Greece; 9 Qingdao Innovation and Development Center, Harbin Engineering University, Qingdao 266000, China

**Keywords:** 2D materials, monolayer WS_2_, mechanical strain, spin polarization, photoluminescence, Raman spectroscopy

## Abstract

Nanoscale-engineered surfaces induce regulated strain in atomic layers of 2D materials that could be useful for unprecedented photonics applications and for storing and processing quantum information. Nevertheless, these strained structures need to be investigated extensively. Here, we present texture-induced strain distribution in single-layer WS_2_ (1L-WS_2_) transferred over Si/SiO_2_ (285 nm) substrate. The detailed nanoscale landscapes and their optical detection are carried out through Atomic Force Microscopy, Scanning Electron Microscopy, and optical spectroscopy. Remarkable differences have been observed in the WS_2_ sheet localized in the confined well and at the periphery of the cylindrical geometry of the capped engineered surface. Raman spectroscopy independently maps the whole landscape of the samples, and temperature-dependent helicity-resolved photoluminescence (PL) experiments (off-resonance excitation) show that suspended areas sustain circular polarization from 150 K up to 300 K, in contrast to supported (on un-patterned area of Si/SiO_2_) and strained 1L-WS_2_. Our study highlights the impact of the dielectric environment on the optical properties of two-dimensional (2D) materials, providing valuable insights into the selection of appropriate substrates for implementing atomically thin materials in advanced optoelectronic devices.

## 1. Introduction

Transition Metal Dichalcogenides (TMDs), MX_2_ (M = Mo, W, and X = S, Se, Te), are layered semiconductors with a crystal structure similar to graphene. Their interlayer bonding is characterized by weak van der Waals forces, enabling the isolation of atomically thin TMD layers. Unlike graphene, TMDs have a finite energy bandgap, making them excellent candidates for optoelectronic devices [1,2,3,4]. Monolayers of such materials undergo a transition from indirect to direct bandgap semiconductor at the K point of the Brillouin Zone (BZ), leading to the formation of stable excitons even at room temperature with very high binding energies (hundreds of meVs) [5,6]. In addition, because of the broken inversion symmetry, strong spin–orbit interaction, and time reversal symmetry, they have unique valley-dependent optical selection rules [7]. The inherent coupling of the valley and spin indices makes the valley index a potential new degree of freedom that can be addressed using helicity-resolved excitation [7,8,9,10,11]. As membranes, they are susceptible to their surrounding dielectric environment and to their supporting substrate [12,13,14]. Specifically, engineered substrates can significantly affect the intensity of the emitted photoluminescence (PL) as well as the degree of valley polarization (VP). For instance, reports show that 1L-WSe_2_ on hybrid plasmonic nanostructures and/or nano-antennas presents giant PL enhancement [15,16], while others reveal that coupling with Bragg gratings can show record valley contrast at room temperature [17]. In addition, transferring monolayer TMDs onto nano-pillars is associated with quantum emission, resulting from the funneling and confinement of excitons at their apex [18,19]. Another approach to tailoring the optical properties of 2D materials aiming for flexible electronics is mechanical strain [20,21,22]. Controlled deformation of monolayers via strain (uniaxial and/or biaxial) can directly affect the band structure of TMDs and enable control of their optical properties [23,24,25,26,27,28]. Any supporting substrate alters the band structure of atomically thin materials due to lattice mismatch or local fluctuations in the dielectric constant [14,29]. Furthermore, trapped moisture between the substrate and the flake can affect PL efficiency and linewidth, as previously reported [14]. Thus, understanding the optical response of a free-standing 2D material is crucial for devices.

Here, we follow an all-dry technique to transfer WS_2_ monolayers exfoliated from bulk crystals over pre-patterned Si/SiO_2_ substrates with cylindrical wells, using viscoelastic stamping [30]. Upon fabrication, we follow a simple, controlled, and reproducible method to identify three different areas on the same flake (suspended, supported, and fully conformed), which can be identified based on the different optical contrast and morphology. We combine Scanning Electron and Atomic Force Microscopies (SEM, AFM) with PL and differential reflectivity (DR/R) to link the morphological differences with the corresponding optical response of the 1L-WS_2_. Interestingly, temperature-dependent helicity-resolved PL studies show that suspended 1L-WS_2_ preserves circular dichroism in the 150–300 K temperature range. We attribute this result to increased absorption in these areas, which arises from the coincidence of the excitation energy (2.28 eV) with the blue-shifted excited A:2s state of 1L-WS_2_. Our studies demonstrate the effect of the dielectric environment on a single 2D TMD flake, paving the way for topological tailoring of its optical properties by selecting appropriate substrate configurations. This opens avenues for the development of 2D material optical devices. Patterned substrates can enhance light–matter interactions, leading to improved efficiency in solar cells and sensors. Additionally, the ability to control excitonic properties through strain or patterning can lead to the development of advanced quantum devices, such as single-photon emitters for quantum computing and secure communication technologies, photodetectors, and LEDs.

## 2. Materials and Methods

### 2.1. Substrate Patterning and Sample Preparation

**Figure 1:**
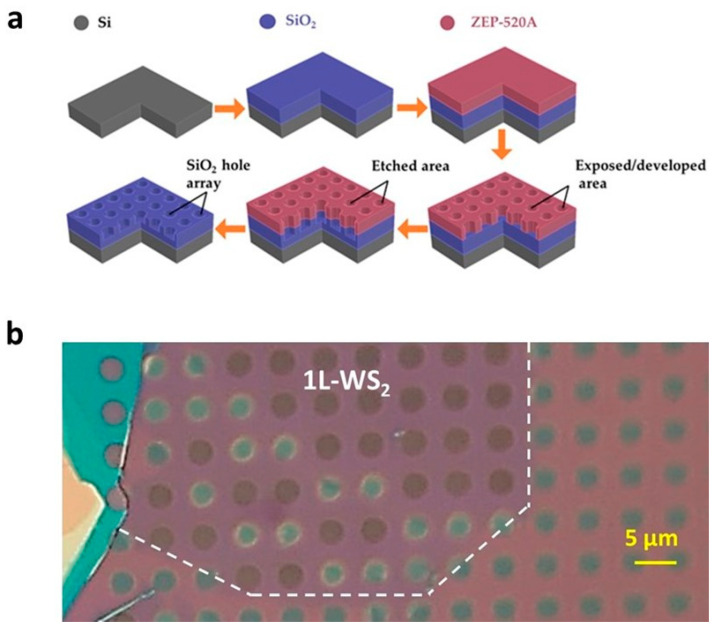
(**a**) Schematic of the SiO_2_ cylindrical well arrays and overall fabrication steps. The well diameter ranges from 1 to 5 microns, and the period ranges from 3 to 8 microns. The depth (174 nm) was defined by the etching time. (**b**) Optical image of a WS_2_ crystal transferred onto the substrate in (**a**) with a well diameter of 3 μm. The monolayer WS_2_ is delineated by the dashed white curve where “dark” and “bright” wells are clearly discriminated.

A single-polished p-doped (100)-oriented 4-inch silicon wafer (525 µm thick, resistivity of 0.8–1.2 Ωcm) was subjected to ultrasonic-assisted organic cleaning using acetone for 5 min, rinsed with isopropanol, followed by native oxide removal in a solution of BOE 1:6 for 2 min (Figure 1a). A thin film of SiO_2_ was then immediately deposited using an Oxford PlasmaPro 100 plasma-enhanced pressure chemical vapor deposition (PECVD). The process used the reaction of silane (SiH_4_) and nitrous oxide (N_2_O) gases, the ratio of which determined the index of refraction. The deposition took place at a platen temperature of 350 °C and a pressure of 12 mTorr with gas flow rates of SiH_4_ at X and N_2_O at Y standard cubic centimeters per minute (sccm). The thickness and index of refraction were measured by a J.A. Woollam M-2000 ellipsometer (J.A. Woollam Co., Inc., Lincoln, NE, USA) and verified to be 288 nm and 1.468, respectively. Prior to resist coating, the SiO_2_ surface was treated with hexamethyldisilane (HMDS) to promote adhesion. The sample was then spin-coated with ZEP-520A (positive electron beam resist, Zeon Specialty Materials Inc., San Jose, CA, USA,) at a final speed of 3000 rpm for 50 s. ZEP-520A is a positive e-beam resist, consisting of copolymers of a-chloromethacrylate and a-methylstyrene that has high resolution, sensitivity, and contrast, as well as relatively good dry etch resistance.

The sample was baked at 180 °C on a hot plate for 3 min. By using this recipe, homogeneous resist coating with a thickness of around 400 nm was obtained. The direct electron beam writing was carried out using a RAITH Voyager e-beam lithography (EBL) system (RAITH GmbH, Troy, NY, USA ) at a constant acceleration voltage of 50 kV and emission current of 10 nA. The exposure dose varied in the range of 200–280 μC/cm^2^. After the exposure, the pattern was developed in ZED-N50 for 90 s, followed by a rinse in a solution of MIBK/IPA of 9/1 for 60 s, and finally, blow-dried using N_2_. The pattern was transferred into the SiO_2_ by a dry etching process using an Oxford PlasmaLab 100 inductively coupled plasma reactive ion etching (ICP-RIE, Oxford Instruments America Inc., Concord, MA, USA) at a constant temperature of 20 °C and pressure of 20 mTorr. The gases utilized were CHF_3_ and Ar at constant flow rates of 12 sccm and 38 sccm, respectively, and the forward RIE bias power was set at 200 watts. The etching times were varied in the range of 250 to 350 s to acquire the desired depth of 175 nm. After etching, the residue of the ZEP-520A resist on the sample was removed using the Plasma-Preen Plasma Cleaning/Etching System (Plasmatic Systems, Inc., North Brunswick, NJ, USA). The patterned structure was evaluated using ZEISS Ultra-55 Scanning Electron Microscope (Carl Zeiss Microscopy, LLC, White Plains, NY, USA). Finally, for convenience, the back side of the wafer was scribed into 1 × 1 cm^2^ chips via an Oxford Laser Dicer E-355 (Oxford Lasers Inc., Shirley, MA, USA).

High-quality WS_2_ bulk crystals (2D Semiconductors) were mechanically exfoliated directly on Polydimethylsiloxane (PDMS) films, which were placed onto typical microscope glass slides. Utilizing a dry transfer protocol [30], including viscoelastic stamping performed by an in-house designed transfer setup. PDMSs were synthesized from a 10:1 mixing ratio (SYLGARD 182 Silicone Elastomer Kit) (Dow Chemical Company, Midland, MI, USA) utilizing heat curing at 80 °C for two hours to achieve preferable elasticity.

Selected single layers were transferred onto a Si/SiO_2_ (288 nm) substrate patterned with cylindrical wells of 3 μm in diameter and 174 nm in depth. The pre-patterned substrate is fixed on a custom hotplate base. Prior to the release step, the temperature is raised to 60 °C, enabling a smoother release of the polymer film. This way, the probability of breaking the monolayer during the process is reduced. A 5 min period was given to the monolayer to create stronger electrostatic forces with the pre-patterned substrate. During this short amount of time, dynamic contrast changes in the optical images could be observed. In particular, upon transferring a monolayer of WS_2_ onto the pre-patterned substrate, all the cylindrical wells beneath the flake appeared “dark” due to optical interference effects. Over the course of a 5 min waiting time, the contrast in several wells underwent a transition from “dark” to “bright” as air was released (Figure 1b). Although this transition did not occur simultaneously in all wells, it was consistently observed across all transfer processes.

### 2.2. Photoluminescence, Reflectivity, and Photoluminescence Excitation Spectroscopy

Differential reflectivity (DR/R), micro-PL (μPL), and spin–valley polarization studies were carried out in a custom optical setup. A typical CW He-Ne 543 nm laser was used as excitation source. Polarization measurements were performed following a slightly modified optical path incorporating a quarter-wave plate to induce circular polarization on the excitation laser. DR/R experiments were performed using a supercontinuum light (360 nm–2600 nm) of a typical tungsten-halogen source (Thorlabs SL201L, Thorlabs, Dortmund, Germany). A Janis ST500 cryostat (Lake Shore Cryotronics, Westerville, OH, USA) was used for temperature-dependent studies. All signals were analyzed in an iHR-320 spectrometer (Horiba Scientific, Longjumeau, France). For Photoluminescence Excitation Spectroscopy (PLE) measurements, a supercontinuum white light (SuperK EVO HP EU-4, NKT Photonics, Birkerod, Denmark) was coupled with a SuperK VARIA filter and a SuperK Connect (FD2 non-PM) broadband fiber delivery. The white light was transmitted through the SuperK VARIA filter, which functioned as grating and analyzed the light, enabling the selection of specific wavelengths within the visible spectrum with nanometer precision. The chosen excitation wavelength was then conveyed to the experimental setup via an optical fiber.

### 2.3. Atomic Force Microscopy (AFM)

AFM imaging has been carried out in intermittent contact mode in ambient conditions. Instrument Bruker (model: Dimension Icon) was used for scanning the silicon nitride probe (Kn = 04 N/m, diameter around 20 nm). The operation is carried out in the insulation box and over the anti-vibrant stage to minimize the building and external vibrations. The calibration of the cantilever is carried out through thermal tuning, and Sader’s method is described in detail elsewhere [31].

### 2.4. Raman Spectroscopy

Raman spectra were collected with a Renishaw inVia Raman spectrometer (Renishaw, UK) in the backscattering geometry. The beam of a solid-state 515 nm laser was focused by means of a 50× objective lens (N.A. = 0.75). Laser power was kept below 0.1 mW to avoid laser heating effects and photodoping. The Raman scattered radiation was dispersed by 2400 grooves/mm diffraction grating. Our system uses a Renishaw MS100 (Renishaw, Gloucestershire, UK) encoded motorized XYZ sample stage, allowing collection of Raman maps with a step size of 100 nm.

## 3. Results and Discussion

**Figure 2:**
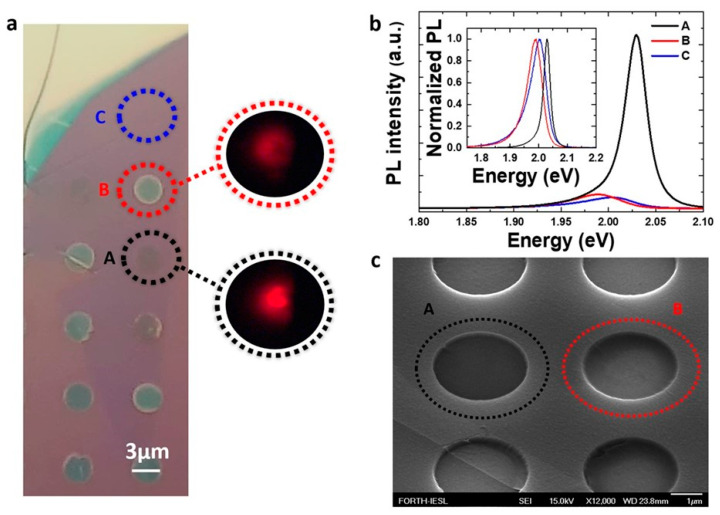
(**a**) Optical image of a part of the monolayer WS_2_ over the substrate shown in Figure 1b, A: dark wells, B: bright wells, and C: simply supported. The circles correspond to a PL intensity map collected from “A” and “B” wells. (**b**) Room temperature PL spectra of the corresponding areas “A”, “B”, and “C” in (**a**). (**c**) SEM image of a part of the transferred monolayer, including areas “A” and “B”.

Following the transferring protocol described in Section 2.1, “dark” and “bright” holes are established in single-layer (1L) WS_2,_ as shown in Figure 2a. Area “C” corresponds to supported 1L laying on Si/SiO_2_ (288 nm), whereas areas “A” and “B” correspond to 1L on optically contrasted “dark” and “bright” wells, respectively (Figure 2a). These optical-contrast differences (“dark” vs. “bright”) are correlated directly to the dramatic difference in the emitted PL intensity. Figure 2b shows μ-PL spectra at 300 K from the three adjacent areas. A 10-fold enhancement in PL efficiency and a 30 meV blue-shift in emission energy is observed in spectra collected from area “A” with respect to area “C”. This emission energy change is expected, as the part of the sample in area “C” is lying on the substrate and may not be entirely flat. Additionally, the PL linewidth from area “C” is broader compared to area “A”, possibly due to trapped moisture or molecules in the interface of the substrate, impurities, and/or local field fluctuations (substrate carrier doping). These factors can affect the Coulomb interaction, leading to inhomogeneous broadening [14,29]. In area “B” (“bright” well), there is no significant enhancement in the PL signal, and the emission energy is slightly red-shifted (1.98 eV) compared to the reference supported area “C” (2.00 eV). This red-shift is a clear indication of mechanical strain and will be discussed later. Figure 2c presents an SEM image of two neighboring wells covered with 1L-WS_2_, exhibiting different optical contrasts similar to those observed in microscopy images (Figure 1b and Figure 2a). This clear difference can be attributed to the form the 1L is conformed onto the wells at “A” and “B”. In the SEM image shown in Figure 2c, the boundary of the 1L-WS₂ monolayer is clearly discernible. The image reveals that half of the cylindrical well is covered by a 1L-WS_2_, which conforms closely to the surface of the well. This coverage highlights the 1L’s continuous spread across the substrate and its uniform adherence to the underlying surface.

Apparently, during the fabrication process (Section 2.1), the presence or absence of trapped air in the wells may account for the formation of a suspended 1L-WS_2_ or a fully conformed onto the well topography, respectively. However, a more direct approach is needed to support this identification.

**Figure 3:**
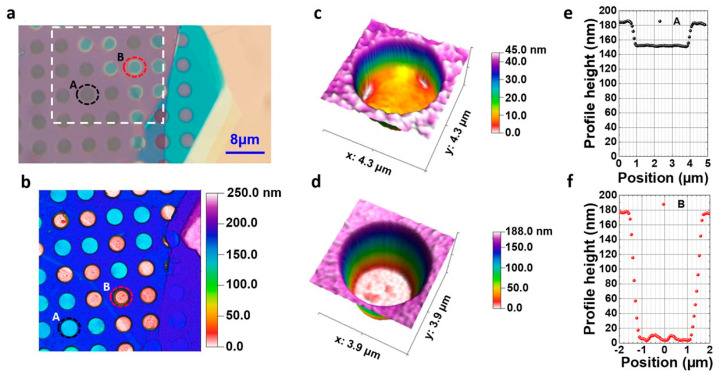
(**a**) Optical image of the selected areas (“A” and “B”) for the AFM experiment. (**b**) Depth color map extracted by AFM. (**c**,**d**) 3D topography of the “A” and “B” wells, respectively. (**e**,**f**) Height profiles of the “A” and “B” wells along a diameter.

Using AFM, we scanned the area enclosed in the dashed rectangular shown in Figure 3a, resulting in a topography map shown in Figure 3b. This map highlights the contrast differences identified in both optical microscopy and SEM images. In particular, the light blue circles (e.g., A) and pink (e.g., B) wells correspond to the “dark” and “bright” contrast wells, respectively, indicating the difference in the actual depth between them. Focusing on the two wells denoted as “A” and “B” in Figure 3b, their 3D topography is illustrated in Figs 3c and 3d, respectively. The corresponding height profiles (Figure 3e,f) along a diameter in each well revealed depths of about 30 nm for well “A” and 180 nm for well “B”, indicating a suspended in “A” and a fully conformed 1L-WS_2_ in “B”. The fluctuations observed at the bottom of well “B” (Figure 3d,f) are attributed to the amorphous nature of the SiO_2_ substrate. In conclusion, the “dark” and “bright” wells observed in the microscopy image (Figure 3a) correspond to suspended and fully conformed monolayers, respectively.

**Figure 4:**
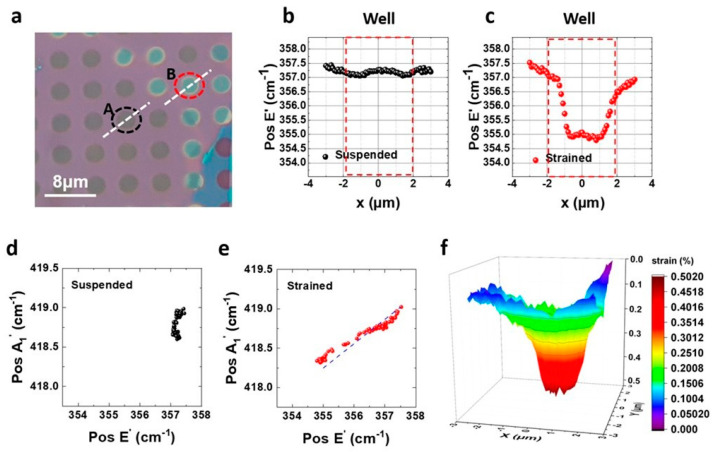
(**a**) Optical image of the selected areas for Raman mapping. (**b**,**c**) Energy of E^’^ mode with respect to lateral dimensions over the well for areas “A” and “B”, respectively. (**d**,**e**) Pos(A_1_’)—Pos(Ε’) correlation plots for the “A” and “B” regions. (**f**) Three-dimensional color map of strain value for area “B”.

The shape of the monolayer in each well, as revealed by AFM, results in mechanical deformation and subsequent strain development. To quantify the strain, we conducted Raman mapping experiments. For consistency, we collected Raman signal along a diameter (Figure 4a) of the same wells “A” and “B”, which were studied with AFM. The most prominent peaks in the 250–450 cm⁻^1^ spectral region of WS_2_ are the E_2g_^1^ (E’ in monolayer) and A_1g_ (A₁’ in monolayer) modes, corresponding to in-plane and out-of-plane vibrations of W and S atoms, respectively. Along with these, the Raman spectrum of WS_2_ shows other strong peaks, such as the 2LA mode, which represents higher-order vibrational modes. Notably, the 2LA and E’ peaks are closely spaced. While E’ mode is sensitive to strain or defects, the A₁’ mode is very sensitive to the number of layers and red-shifts as thickness is reduced. The energy difference of approximately 61 cm^−1^ between the two main vibrational modes E’ and A_1_’ is the signature of monolayer thickness (see Supplementary Material, Figure S1). Additionally, for a WS_2_ monolayer, the 2LA mode has about twice the intensity of the first-order A_1_’ peak when the excitation laser is 514 nm [32].

In Figure 4b,c, we initially plot the peak position, Pos(E’), of the strain-sensitive in-plane Raman mode E’ of 1L as a function of the distance from the center (x = 0 μm) of the well [27]. In Figure 4b, we identify a slight fluctuation of Pos(E’) around 357.3 cm^−1^ within the well, while in Figure 4c, Pos(E’) shifts to lower values and shows slight fluctuation around 355 cm^−1^ within the well. We can thus conclude that for well A, there is no significant shift of E’ mode and, therefore, negligible strain associated with the monolayer in suspension. In contrast, for well “B”, we observe a red-shift of the Pos(E’) of about 2.5 cm^−1^ relative to the mean value of Pos(E’) of simply supported 1L, indicating significant strain with its maximum value at the center of the well (Figure 4c). To support that, we make use of the Pos(A_1_’)—Pos(Ε’) plot for the “bright” and “dark” regions [33]. The correlation data points relevant to the suspended region form a well-defined cluster (Figure 4d) with minimal frequency changes for the A_1_’ and E’ peaks of less than 0.5 and 0.2 cm^−1^, respectively. On the other hand, in the conforming region (region “B”), the data points show a well-defined linear correlation (Figure 4e). In the same plot, the dashed blue line indicates the linear correlation of a Pos(A_1_’)—Pos(Ε’) correlation plot from a biaxially strained WS_2_ monolayer [27]. Since the slopes of these two traces match, we can conclude that the peak frequency shifts in area “B” are predominantly due to changes in mechanical strain. The measured frequency shift of the E’ mode at the center of the well corresponds to a biaxial strain value of approximately 0.5%.

**Figure 5:**
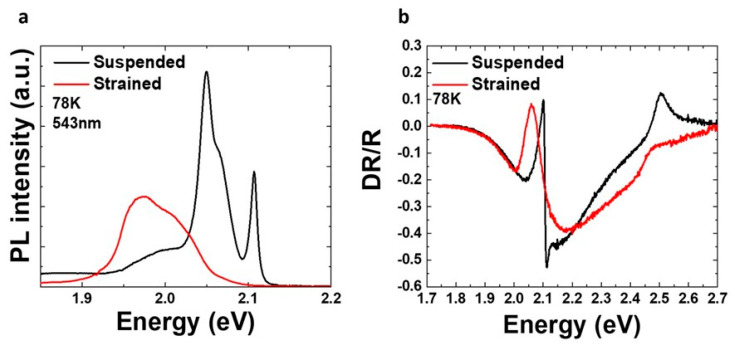
PL (**a**) and differential reflectivity (**b**) of suspended (black line) and strained (red line) 1L at 78 K.

Having clear evidence of the conformation morphology of the 1L over the pre-patterned Si/SiO_2_ substrate, we investigated the optical properties of the suspended and strained areas at low temperatures. Figure 5a shows the PL spectra at 78 K of suspended (black line) and strained (red line) regions, excited with a 543 nm continuous-wave laser. In regions where the monolayer is suspended over the wells, the spectra are well defined, and the excitonic transitions are clearly resolved. The PL peak at 2.10 eV is assigned to the light emission from the neutral excitons (X^0^). This is verified by the differential reflectivity measurements shown in Figure 5b, where the derivative-like shape transition (black line) at 2.1 eV is due to neutral excitons, whose oscillator strength is greater than that of the charged excitons. The integrated PL intensity of excitons (X^0^) from the suspended regions is significantly stronger compared to both the strained regions (Figure 5a) and the regions supported on Si/SiO_2_ (Supplementary Material Figure S2). The suspension of the 1L-WS_2_ suppresses the disorder caused by the substrate, leading to a more efficient radiative recombination. At lower energies, there is a convolution of two peaks. The shoulder peak at 2.068 eV is from charged exciton emission (X^−^), whereas the most prominent peak at 2.049 eV can be assigned to localized excitons. In the case of the strained regions, we observe a much broader PL emission (red line) without clearly resolved peaks. After deconvoluting the spectra (see Supplementary Material, Figure S3), the emission peak from the neutral excitons (X^0^) is found at 2.061 eV, almost 40 meV lower than the corresponding one at 2.1 eV in the suspended areas. The PL intensity in this case is significantly lower, mainly due to mechanical strain and disorder induced by the substrate. Comparison of the PL intensity ratio of neutral to charged excitons ($\frac{I^{X^{0}}}{I^{X^{-}}}$ = 1.3 or 0.12 for the suspended and strained regions, respectively) shows a significant reduction in electron density in the suspended flakes. This order of magnitude difference is attributed to the substrate disorder and the efficient conversion of excitons to trions (and bound excitons) induced by strain [28].

**Figure 6:**
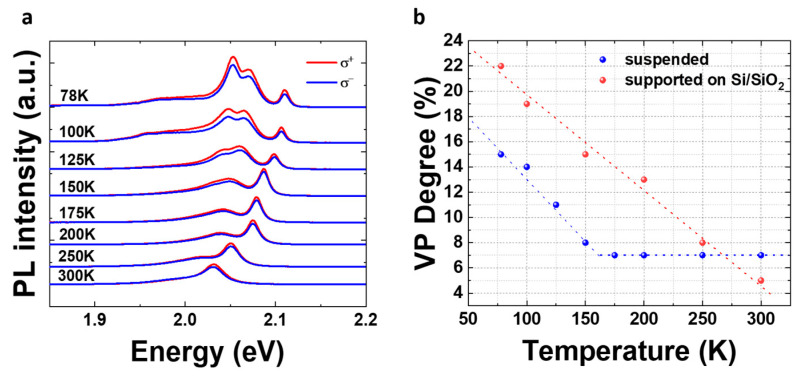
(**a**) Temperature-dependent spin–valley polarization measurements of a characteristic suspended area. (**b**) Degree of valley polarization as a function of temperature for a suspended (blue dots) and supported (red dots) flake.

To evaluate the effect of suspension on the degree of valley polarization of 1L-WS_2_, we performed temperature-dependent spin–valley polarization measurements. The circular polarization (valley polarization, VP) is determined by the ratio

$$\mathrm{VP}=(I_{\sigma +}-{Ι}_{\sigma -})/(I_{\sigma +}+{Ι}_{\sigma -})$$

where $I_{\sigma +}$ and $I_{\sigma -}$ denote the right-/left-handed polarization resolved PL intensity, respectively. In the following, we focus only on the neutral exciton emission. Figure 6a shows the PL spectra from 78 K to 300 K analyzed in σ^+^ (red) and σ^−^ (blue) for the suspended part of the sample. After 150 K, the PL emission is dominated by neutral excitons.

In Figure 6b, the temperature-dependent VP degree (%) for suspended 1L regions is presented. These data were taken using an off-resonance excitation wavelength (543 nm CW laser). The circular polarization depends on the effective exciton lifetime (τ_e_) and the valley relaxation time (τ_v_) through the relation $P_{0}/(1+\frac{\tau_{e}}{\tau_{v}})$, where P_0_ is the initial polarization [34]. The effective exciton relaxation time includes both radiative and non-radiative processes. At 78 K, we measure a ~15% valley polarization, which is considerably lower than the ~22% for a 1L on Si/SiO_2_ under the same experimental conditions. The reduced VP in the suspended part of the sample may be due to the suppressed disorder from the substrate. Minimized disorder is linked with a decreased number of scattering channels, which can lead to longer non-radiative lifetimes for the excitons and, by extension, a reduced degree of VP [35]. In the 78–150 K temperature range, polarization drops similarly for both suspended and supported 1L-WS_2_. However, this is not the case for higher temperatures. The degree of VP drops for the supported 1L with the same rate for all temperatures up to 300 K, where it almost vanishes. This result is expected since we pump the system with energies exceeding 190 meV above the 1s exciton state. On the other hand, at 150 K, the VP of suspended 1L-WS_2_ reaches the value of 7%, which is preserved up to room temperature, forming a plateau (Figure 6b). 

**Figure 7:**
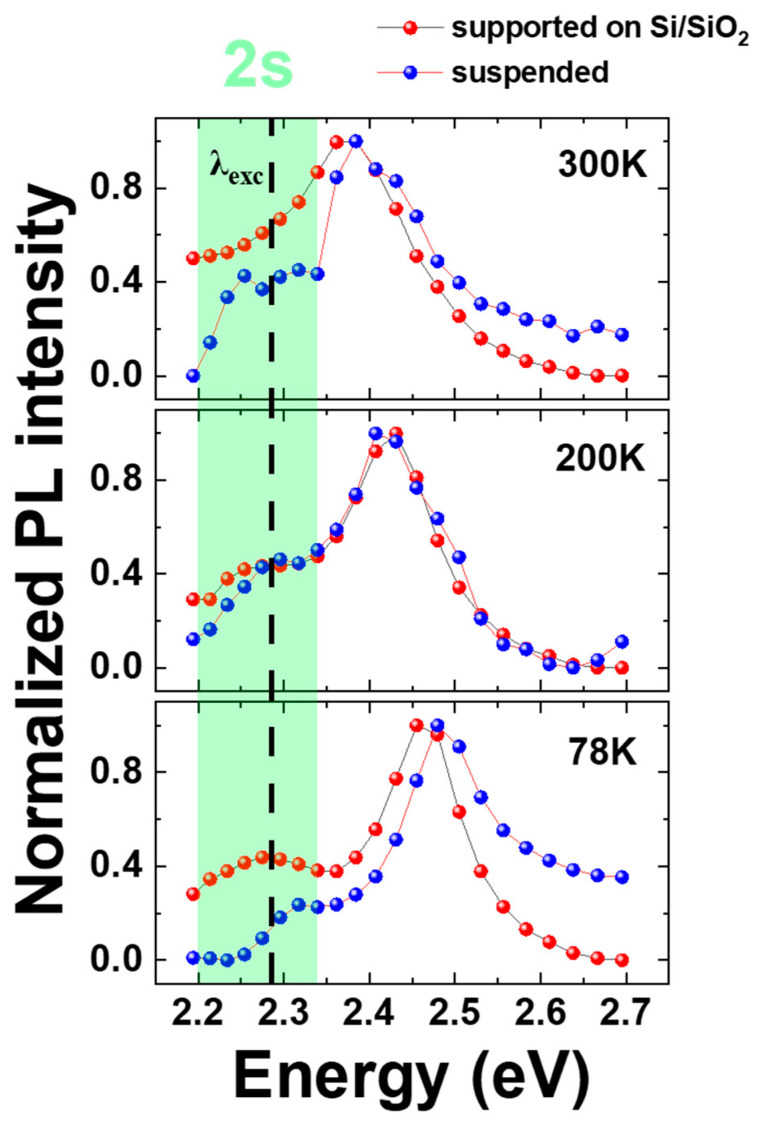
Comparative study of PLE spectra for suspended 1L-WS_2_ (blue spheres) and supported 1L-WS_2_ (red spheres) in 78 K, 200 K, and 300 K. Green area represents the energy range for the A:2s state.

To explore the origin of this plateau in the case of the suspended part of the sample, we performed Photoluminescence Excitation Spectroscopy measurements (PLE). Specific positions on the sample were selected (supported and suspended regions), and we monitored the normalized PL intensity while changing the excitation wavelength. We aimed to investigate the possibility of blue-shifted excited states for suspended 1L-WS_2_ falling in resonance with the excitation energy. Indeed, in Figure 7, we observe a signature for blue-shifted energy for the A:2s state in suspended 1L. Considering this energy blue-shift of the excited state and the fact that our pump energy is fixed at 2.28 eV, we can confidently state that in the temperature range of 150–300 K, excitation energy will be on resonance (and/or near resonance) with the A:2s state of suspended 1L. This effect will trigger enhanced absorption in suspended monolayer areas [36]. The strong absorption will follow an efficient relaxation to the A:1s state through the emission of phonons [37]. A number of prominent phonons have been reported for single-layer WS_2_ (and other monolayer TMDs) through Raman studies [38,39]. This relaxation mechanism preserves valley polarization and valley coherence [36] and, thus, can explain why we obtain a non-zero VP up to room temperature despite pumping the system approximately 190 meV above the emission of X^0^.

## 4. Conclusions

In summary, we studied the impact of substrate-induced disorder on the optical properties of 1L-WS_2_ by transferring the monolayer onto a pre-patterned Si/SiO_2_ substrate with cylindrical wells, thereby creating regions where the monolayer is either suspended and strain-free or conformed and under strain. Our method enabled us to produce both types of regions on the same flake through a straightforward transfer process. AFM and SEM microscopy confirmed the successful conformation of 1L-WS_2_ over the patterned substrate. Raman mapping verified the creation of suspended areas and revealed the presence of mechanical strain in the fully conformed 1L-WS_2_ regions within the cylindrical wells on the same flake. Optical spectroscopy (PL and Reflectivity) further demonstrated the effects of substrate disorder on PL intensity and linewidth. This is clearly evidenced by comparing the intensity ratios of X^0^/X^−^ for the suspended and strained (or supported) areas at 78 K. Additionally, helicity-resolved PL experiments revealed a non-zero plateau of VP maintained up to 300 K in the suspended 1L-WS_2_. This observation is attributed to a resonance between the excitation energy (2.28 eV) and the blue-shifted excited A:2s state of suspended 1L-WS_2_ in the 150–300 K temperature range. The enhanced absorption and efficient relaxation back to A:1s via phonon interactions are responsible for the preserved degree of VP. Our work provides valuable insights into the topological conformation of a 2D TMD on a patterned substrate and highlights the significant influence of the dielectric environment on its optical properties. This underscores the importance of selecting an appropriate hosting substrate for 2D materials applications. Suspending the 2D material is preferable for enhancing the PL intensity since it minimizes the substrate-induced disorder. Conversely, full conformation to the substrate shape induces an appreciable amount of strain that tunes the PL emission energy, aligning with the broader research area of straintronics.

## Data Availability

The data that support the findings of this study are available within the article and its Supplementary Materials.

## Supplementary Materials

The following supporting information can be downloaded at https://www.mdpi.com/article/10.3390/nano14171437/s1. Figure S1: Raman spectrum taken at T = 300 K with 514 nm excitation wavelength from area “C”—1L-WS_2_ simply supported. Deconvoluted was employed to determine the main vibrational modes: E’-in plane mode, A1’-out of plane mode, and 2LA-second order longitudinal acoustic mode at the M point of the Brillouin zone. Figure S2: Photoluminescence spectra taken at 78 K with 543 nm excitation wavelength from 1L-WS_2_ from the suspended part (a) and from the supported part of the flake (b). X^0^ and X^−^ indicate the energy position for the neutral and charged excitons, respectively. Figure S3: Deconvolution of PL spectra for the strained and suspended areas of 1L-WS_2_ at 78 K.
