# Supplementary material for: Texture-Induced Strain in a WS2 Single Layer to Monitor Spin–Valley Polarization
Source: Nanomaterials (Basel). 2024 Sep 3;14(17):1437. doi: 10.3390/nano14171437 (PMC11397293; doi:10.3390/nano14171437)
Supplement: Supplementary file 1 [file nanomaterials-14-01437-s001.zip › nanomaterials-3170392-supplementary.pdf]

Supplementary Information

Textured-induced strain in a WS_2 single-layer to monitor spin-valley polarization

George Kourmoulakis[1,2], Antonios Michail[3,4], Joseph A. Christodoulides[5], Manoj Tripathi[6], Alan B. Dalton[6], John Parthenios[4,*], Konstantinos Papagelis[4,7,*], Emmanuel Stratakis[1,8,9], and George Kioseoglou[1,2,*]

[1] Institute of Electronic Structure and Laser, Foundation for Research and Technology - Hellas, Heraklion, 71110, Crete, Greece; geokourm@iesl.forth.gr; stratak@iesl.forth.gr; gnk@materials.uoc.gr;

[2] Department of Materials Science and Technology, University of Crete, Heraklion, 70013 Crete, Greece; geokourm@iesl.forth.gr; gnk@materials.uoc.gr

[3] Department of Physics, University of Patras, Patras, 26504, Greece; antmichail@upatras.gr

[4] FORTH/ICE-HT, Stadiou str Platani, Patras 26504 Greece; antmichail@upatras.gr; jparthen@iceht.forth.gr; kpapag@physics.auth.gr

[5] Naval Research Laboratory, 4555 Overlook Ave SW, Washington, DC 20375-5320, U.S.A; joseph.christodoulides@nrl.navy.mil

[6] Department of Physics and Astronomy, University of Sussex, Brighton BN1 9RH, United Kingdom; M.Tripathi@sussex.ac.uk

[7] School of Physics, Department of Solid-State Physics, Aristotle University of Thessaloniki, Thessaloniki,54124, Greece; kpapag@physics.auth.gr

[8] Department of Physics, University of Crete, Heraklion Crete 70013, Greece; stratak@iesl.forth.gr

[9] Qingdao Innovation and Development Center, Harbin Engineering University, Qingdao 266000 Shandong, P.R. China; stratak@iesl.forth.gr

*Correspondence: gnk@materials.uoc.gr ; tel.: +30 2810394318, jparthen@iceht.forth.gr, tel: +30 2610965277, kpapag@physics.auth.gr, tel: +30 2310998031

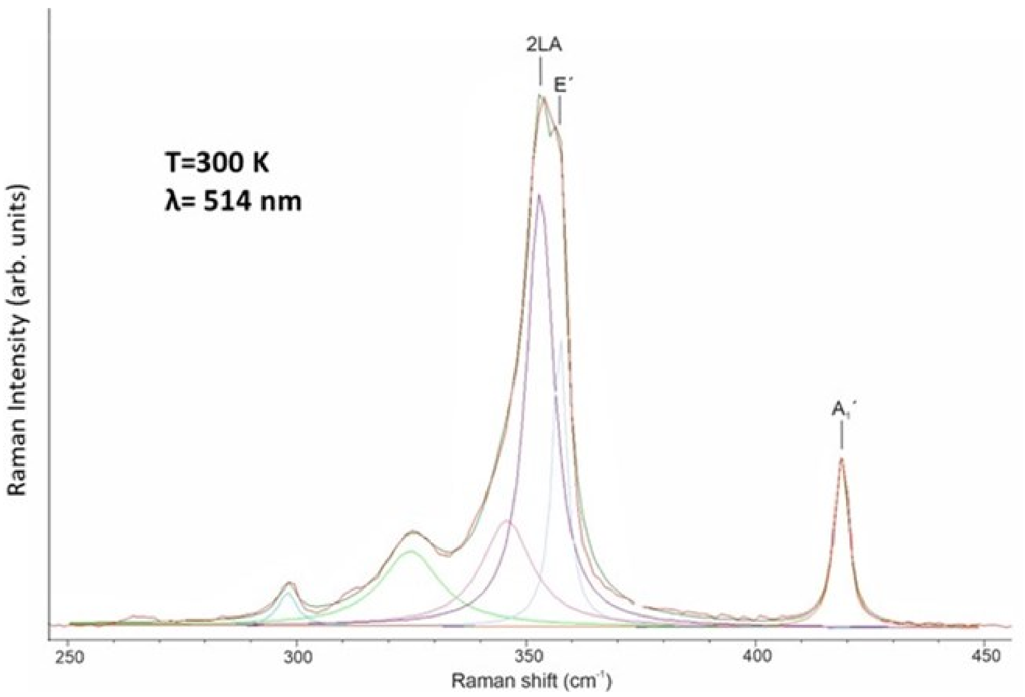

Figure S1: Raman spectrum taken at T=300 K with 514 nm excitation wavelength from area "C" - 1L-WS_2 simply supported. Deconvoluted was employed to determine the main vibrational modes: E′-in plane mode, A1′-out of plane mode, and 2LA-second order longitudinal acoustic mode at the M point of the Brillouin zone.

Figure S1 shows the Raman spectrum taken from the 1L-WS_2, simply supported to Si/SiO_2 substrate (area "C", in Fig.2a of the main text). The most prominent peaks in the 250-450 cm^{-1} spectral region are the E′ and A_1' modes, corresponding to in-plane and out-of-plane vibrations of W and S atoms, respectively. Along with these, the Raman spectrum of WS_2 shows other strong peaks, such as the 2LA mode, which represents higher-order vibrational modes. Notably, the 2LA and E′ peaks are closely spaced. While E′ mode is sensitive to strain or defects, the A_1′ mode is very sensitive to the number of layers and redshifts as this number is reduced. The energy difference of approximately 61 cm^{-1} between the two main vibrational modes E′ and A′ is the signature for monolayer nature. Additionally, for a WS_2 monolayer, the 2LA mode has about twice the intensity of the first-order A_1' peak when the excitation laser is 514 nm [Berkdemir, A., et al. Sci. Rep. **2013**, 3, 1755].

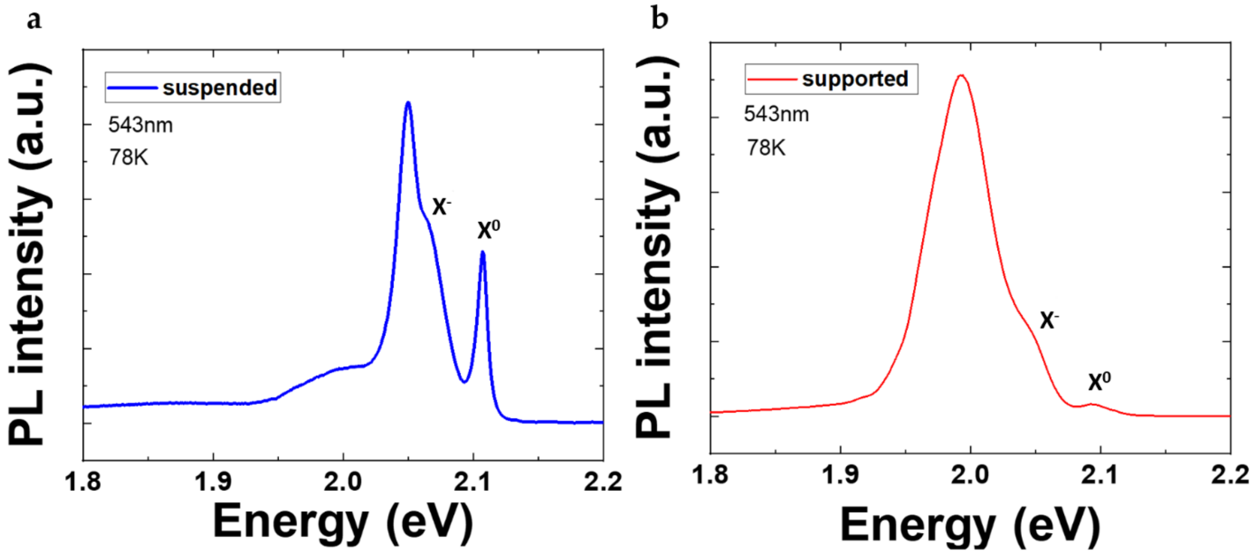

Figure S2: Photoluminescence spectra taken at 78K with 543nm excitation wavelength from 1L-WS_2 from the suspended part (a) and from the supported part of the flake (b). X^0 and X^- indicate the energy position for the neutral and charged excitons, respectively.

In Figure S2, the impact of substrate disorder on the intensity of the neutral exciton (X^0) emission is evident. The intensity ratio between neutral and charged excitons (I_{X^0}/I_{X^-}) is an order of magnitude higher in suspended areas compared to supported areas (and strained areas, see Figures 5a and S2) of the same 1L-WS_2 flake.

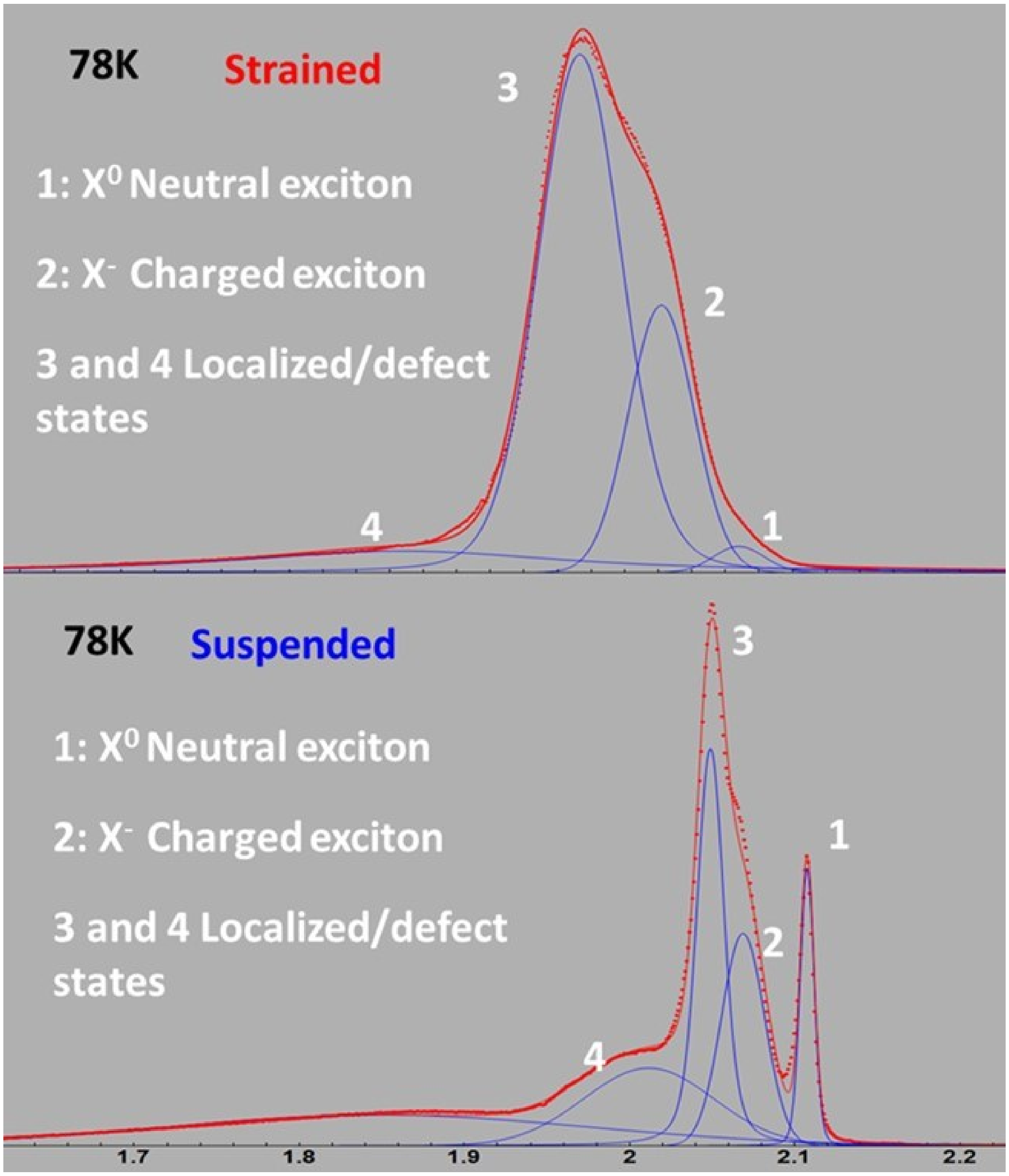

Figure S3: Deconvolution of PL spectra for the strained and suspended areas of 1L-WS_2 at 78K

Deconvolution of low temperature PL spectra was employed to determine the energy position of neutral excitons in strained areas (complementary with Reflectivity, see Figure 5b main text). The results highlight the red shifted energy due to mechanical strain, as well as the prominent difference in intensity of X^0 between suspended and strained areas. The suppressed intensity of X^0 in strained areas stems from substrate disorder and an interplay between neutral and charged/localized excitons.
